# Sustaining the helpers: Mixed methods study of volunteers’ resilience in coastal disaster Java

**DOI:** 10.4102/jamba.v18i1.2086

**Published:** 2026-05-21

**Authors:** Yuli A. Dewi, Koentjoro Soeparno, Pradytia P. Pertiwi, Mizan B.F. Bisri

**Affiliations:** 1 Faculty of Psychology, Universitas Gadjah Mada, Yogyakarta, Indonesia; 2 Faculty of Psychology, Universitas Islam Sultan Agung, Semarang, Indonesia; 3 Graduate School of International Cooperation Studies, Kobe University, Kobe, Japan; 4 Asian Disaster Reduction Center (ADRC), Kobe, Japan

**Keywords:** disaster, volunteer, resilience, coastal, mixed methods

## Abstract

Recurrent climate-driven flooding in the low-lying coastal communities of Demak, Central Java, significantly strains local capacity and tests disaster volunteer resilience. Following two major floods in March 2024, Indonesian Red Cross (PMI) volunteers engaged in a 2-month emergency response. This sequential explanatory mixed methods study investigated the levels of volunteer resilience and the multisystem factors that support them. A survey was conducted with 69 PMI volunteers (aged 18–55 years) using an adapted Connor–Davidson Resilience Scale (CD-RISC), followed by semi-structured interviews with 17 participants, including volunteers, family members and PMI staff. Descriptive statistical and thematic analyses were used, and the findings were integrated through a joint display. Findings indicated that 63.8% of participants exhibited moderate resilience, with personal competence and internal trust showing relatively higher prominence. The qualitative findings identified five themes contributing to disaster volunteer resilience: personal competence, spirituality, cultural values, emotional support and functional support.

**Contribution:**

This study extends disaster risk scholarship by showing that disaster volunteer resilience in a flood-prone coastal setting is sustained through multisystem interactions rather than individual capacity alone. The integration of quantitative and qualitative findings highlights resilience resources that are not fully captured by standard quantitative indicators, including culturally rooted values, familial support, collective spirituality and organisational functional support. These findings provide context-specific insights for strengthening affiliated volunteer support in disaster risk reduction and humanitarian response.

## Introduction

Disaster volunteers play a vital role across the continuum of disaster management, including preparedness, emergency response and recovery. Research on volunteerism in disaster contexts has been documented since the 1950s, and the field has continued to develop, reflecting sustained scholarly attention to volunteers’ roles across different phases of disaster management (Johansson 2024). In many disaster settings, volunteers are among the earliest responders, providing logistical assistance, psychosocial support and other forms of practical help under conditions of uncertainty, urgency and prolonged disruption (Hinsch & Douglas 2024; Kirac, Shaltayev & Wood 2024). As part of the helping workforce, disaster volunteers may face a range of mental health risks, including post-traumatic stress symptoms, anxiety, depression and other forms of psychological distress (Hamblen, Barnett & Norris 2012; Santucci 2012; Shakespeare-Finch & Scully 2009). However, although the burdens experienced by responders have been widely acknowledged, less attention has been given to how disaster volunteer resilience is conceptualised and sustained, particularly among affiliated volunteers working within established humanitarian organisations (Kesgin & Durmuş 2022). This issue is particularly relevant in climate-vulnerable coastal settings, where disaster response often takes place under recurrent and prolonged adversity (Islam et al. 2021; Sengupta 2023). International research on climate change impacts in coastal areas has grown rapidly, with increasing attention to hydrometeorological hazards such as tidal flooding, land subsidence and repeated inundation (Laino & Iglesias 2023; Mohan, Kumari & Kaushik 2024). Coastal floods in low-lying areas can produce cumulative ecological, social and economic disruption over time (Diem, Minh & Diep 2015; Swarnokar et al. 2025; Thi Phuong et al. 2023). Indonesia is a country consisting of thousands of islands, with the second longest coastline in the world. In addition, its tropical climate gives rise to the threat of flooding because of high rainfall and subsidence that results in tidal floods. Many areas in Indonesia are vulnerable to flooding in coastal areas, such as Jakarta (Bennett et al. 2023), Semarang and Demak in Central Java (Iswari, Prabowo & Yulianto 2025; Wurjanto, Mukhti & Wirasti 2017), Banyuasin District in Sumatra (Hamdani et al. 2014) and Mataram in Lombok (Rahadiati, Munawaroh & Suryanegara 2021). In Central Java, floods have serious implications for the community’s livelihood and environmental security, including damage to agricultural and food security (Suryanto et al. 2023). Severe flooding in coastal areas of national spiked a national concern, citing that the February-March 2024 Demak Regency, Central Java, recorded as the worst flood in the last 30 years. This flood caused economic losses of more than IDR 2.049 trillion, affecting 13 from 14 subdistricts and causing over 56 000 victims (BPBD Kabupaten Demak 2024). In the flood response effort, the Indonesian Red Cross (PMI) Demak Branch assigned more than 100 personnel. The assistance provided is related to logistics distribution, public kitchens, water and sanitation and health services. More than 50 volunteers were assigned in turn to serve the needs of around 5000 packages per day plus other services (Dinas Komunikasi dan Informatika Kabupaten Demak 2024). In response, records were found on the coordination of assigned volunteers and the psychosocial support of volunteers on duty, which still need to be improved. This shows that we need to improve how volunteers are managed and make sure mental health support is included in plans for dealing with disasters. Volunteer participation in disaster management takes place over a wide spectrum in all pre-disaster risk reduction and post-disaster response and recovery activities. Affiliated disaster volunteers are those who are involved voluntarily in disaster efforts under the umbrella of a charity organisation such as the Red Cross Movement (International Red Cross and Red Crescent Movement), UN agencies, International non-governmental organisation (NGO) and Public Institutions (Kesgin & Durmuş 2022). As the world’s attention to disasters increases, the mental health aspects of the helpers involved in them are also increasing. Volunteers are part of the helpers who are at risk of facing various mental health problems, including post-traumatic stress disorder (PTSD), depression, anxiety and substance use disorder (Hamblen et al. 2012; Santucci 2012; Shakespeare-Finch & Scully 2009). Various mental health risks among volunteers require attention and structured support, but attention to volunteer resilience is still limited compared to reactive interventions (Dewi et al. 2025b). The present study focused on volunteers, unpaid individuals affiliated with non-governmental organisations engaged in disaster management, who dedicate their time and energy to helping others during climate change disasters in the coastal context.

## Literature review

### Conceptualising disaster volunteer resilience

Resilience is a concept with a long and evolving history across disciplines, and its meaning has expanded substantially over time. In psychology, resilience has been conceptualised both as a relatively stable personal quality and as a dynamic process of positive adaptation in the face of significant adversity (Southwick et al. 2014; Windle 2011). Windle (2011), for example, argues that resilience should be understood as the process of effectively negotiating, adapting to or managing significant sources of stress or trauma, supported by assets and resources within the individual, their life and their environment. This perspective is important because it shifts attention away from viewing resilience only as something located ‘inside’ the person and towards the broader conditions, relationships and supports that enable adaptation. Resilience may therefore not carry a single uniform meaning across contexts; rather, it may be understood differently across individuals, families, organisations, societies and cultures and may vary across domains of life (Southwick et al. 2014). This shifting paradigm in resilience is particularly relevant in disaster studies, which move from individual coping capacities to broader systems of support. The development of multisystem resilience contracts expands the emphasis on resilience from internal resilience to interactions between various external systems such as families, organisations and socio-cultural systems (Masten & Motti-Stefanidi 2020; Sanson & Masten 2024; Ungar 2021). In this study, resilience is therefore approached not simply as an individual attribute but also as multisystem process shaped by the interaction of personal, relational, organisational and socio-cultural resources. From this perspective, disaster volunteer resilience cannot be reduced to internal strength alone, because volunteers act within organisational structures, depend on family negotiation and permission and draw meaning from cultural and spiritual resources while carrying out demanding humanitarian roles (Hakkim & Deb 2022; Hechanova & Waelde 2017; Masten & Motti-Stefanidi 2020; Thormar et al. 2013; Ungar 2021). Recent applied work has also begun to conceptualise resilience at the level of organisations and workforces rather than only individuals. The Resilience First/Cranfield model defines organisational resilience through the capacities to anticipate, absorb and adapt and positions workforce as one of five core dimensions alongside social, financial, infrastructure and environmental resilience (Denyer et al. 2024). In this formulation, workforce resilience concerns not only the availability of personnel but also the development and maintenance of knowledge, skills, behaviours, values, health, engagement, motivation and well-being under pressure (Sutliff 2026). Although this framework is organisation centred rather than disaster-volunteer specific, it is relevant to affiliated volunteers because they operate within organisational systems that shape preparedness, coordination, learning and support. Accordingly, this study is grounded in multisystem resilience, while drawing on recent organisational/workforce resilience literature to contextualise the organisational setting of affiliated disaster volunteers. This perspective also has implications for how resilience is assessed. In this study, the Connor–Davidson Resilience Scale (CD-RISC) was used to provide a descriptive profile of volunteer resilience and to identify participants with higher resilience scores for the qualitative phase (Connor & Davidson 2003; Dewi et al. 2024). This quantitative assessment was therefore important for mapping resilience levels and guiding participant selection. At the same time, as Windle (2011) notes, definitions of resilience are closely tied to measurement. While quantitative scores are useful for indicating perceived resilience, they do not fully explain how resilience is built and sustained in context. In disaster volunteer settings, resilience may also depend on relational, organisational, cultural and spiritual resources that are not always fully visible through individual-level quantitative indicators. For this reason, a mixed methods approach was particularly useful, as it allowed resilience scores to be interpreted alongside qualitative accounts of the broader social ecology that supports disaster volunteers. Despite growing interest in resilience, empirical research examining how these different systems interact to support affiliated disaster volunteers remains limited (Dewi et al. 2025c). Existing literature has identified factors related to volunteer resilience (Mao et al. 2020), but much of the available evidence has not sufficiently addressed how resilience is sustained within the everyday social ecology of humanitarian response, particularly in low- and middle-income, climate-vulnerable settings (Nasrullah, Refat & Gustavsson 2025). This gap matters because resilience in such contexts may not follow a single universal pattern; rather, it may be shaped by context-specific configurations of adversity, support and meaning. The present study contributes to this debate by examining disaster volunteer resilience not simply as an individual attribute but also as a multisystem and contextually embedded process in a recurrent coastal flood setting. To address this gap, the present study focuses on affiliated volunteers who were involved in coastal disaster, especially in the 2024 flood response operations in Demak Regency, Central Java. Specifically, this study aimed: (1) to assess the resilience of volunteers who responded to large-scale floods in Demak, Indonesia and (2) to examine the factors contributing to disaster-volunteer resilience in coastal climate-related disasters within Indonesia’s rural context, using the multisystem resilience framework. By integrating quantitative and qualitative evidence, this study seeks to clarify how resilience scores can be interpreted alongside the broader relational, organisational, cultural and spiritual processes through which disaster volunteer resilience is supported in context.

## Research methods and design

### Study design

This study adopted a sequential explanatory mixed methods design in which a quantitative survey was followed by qualitative interviews to explain and elaborate on the statistical patterns (Creswell & Clark 2018; Seesawang, Thongtaeng & Nitirat 2023).

### Research participants and setting

Demak Regency is a low-lying coastal and predominantly rural district in Central Java that is highly exposed to recurrent flooding and tidal inundation, where disaster response is coordinated through local government agencies and the PMI. Disaster volunteers affiliated with PMI Demak Regency and participated in the 2024 flood response were invited to take part in this study. The research employed purposive sampling, engaging volunteers from PMI Demak Branch who were deployed in the 2024 floods. The population consists of 73 participants with 94.5% response rate in participating the quantitative phase (*n* = 69). Most participants were male (75.4%) and belonged to the young adult age group of 18–35 years (68.1%). The majority had completed senior high school or an equivalent level of education (63.8%). The qualitative phase employed purposive sampling to select volunteers with high resilience scores from the quantitative phase who agreed to be interviewed and had not previously attended any formal psychological or psychosocial training related to disaster response. In total, 17 participants were interviewed, comprising eight main participants (volunteers) and nine supporting participants (family members and PMI staff or board members). The qualitative sample size was guided by purposive sampling and thematic saturation to provide explanatory depth, consistent with principles of sequential explanatory mixed methods research (Creswell & Clark 2018; Malterud, Siersma & Guassora 2016).

### Data collection

Data collection took place from February 2025 to June 2025 and consisted of completed questionnaires and audio recordings.

### Quantitative data collection

Volunteer resilience was measured using the CD-RISC, which was tested for reliability and validity using confirmatory factor analysis (CFA) and reliability analysis with Jamovi software (Dewi et al. 2024). The results indicated that 20 of the 25 items met the model fit criteria (comparative fit index [CFI] and Tucker–Lewis index [TLI] > 0.9; RMSEA < 0.08). In this study, resilience levels were categorised as follows: (1) Low: Score < (mean − 1 standard deviation [s.d.]), (2) moderate: (mean − 1 s.d.) ≤ score ≤ (mean ± 1 s.d.) and (3) high: Score > (mean + 1 s.d.).

### Qualitative data collection

Semi-structured interviews were conducted offline at participants’ homes or in mutually agreed locations, lasting approximately 30–45 min. In addition, the Photo Elicitation Interview Technique (Dixon & Mather 1981) was used to facilitate discussion. Example of interview questions is attached.

### Data analysis

#### Quantitative phase

Quantitative analysis was conducted using JASP version 0.19.3.0. The 20-item CD-RISC was scored on a 5-point Likert scale from 0 (not true at all) to 4 (true nearly all the time) and summed to obtain a total resilience score (possible 0–80). The descriptive analysis produced demographic analysis and calculated means, s.d. and score distributions for the total scale as well as each dimension and classified total scores into three categories (low, moderate and high resilience).

#### Qualitative phase

Interview recordings were transcribed and analysed using thematic analysis (Braun & Clarke 2006). The thematic analysis was chosen to relate the themes that emerged, especially with the theoretical framework of multisystem resilience (Masten 2021; Ungar et al. 2021a, 2021b) without aiming to build a theory from scratch. ATLAS.ti software is used to facilitate analysis. The analysis followed Braun and Clarke’s six-phase approach, involving iterative coding, theme development and refinement to enhance analytic rigour and credibility (Braun & Clarke 2006).

#### Mixed method integration

Integration of the quantitative and qualitative strands followed a joint-display technique. The results of the level of resilience and interviews were compared side by side. The integration is at the analysis level that was carried out with the aim of comparing as well as contrasting Qual and Quan strands to check the fit of data and draw meta-inferences (Fetters & Tajima 2022).

### Ethical considerations

Ethical clearance to conduct this study was obtained from the Gadjah Mada University Faculty of Psychology Research Ethics Committee (Ref. No.: 1473/UN1/PS.1/SD/PT.01.04/2025). Prior to data collection, informed consent was obtained in written form from all participants before they took part in the survey and interviews. Participants were informed about the study’s objectives, their right to withdraw at any time without penalty and the intended use of the data. To maintain the confidentiality of the data, several measures were implemented: (1) all personal identifiers were removed from the dataset and replaced with unique alphanumeric codes to ensure anonymity; (2) raw qualitative data, such as interview transcripts, were stored on a secure device accessible only to the primary research team and (3) results are reported in an aggregated or anonymised format to ensure that no individual participant or specific family member can be identified from the findings.

## Results

The integrated findings of mixed method data were presented in Table 3. Table 3 comprises volunteer resilience level and qualitative themes that were joined through analysis of convergence, expansion and discordance.

### Quantitative results: Resilience level

Descriptive analysis showed that the mean total resilience score of volunteers was 47.14 (s.d. = 14.24; possible range 0–80). Based on these scores, participants were classified into three categories: Low (< 32.9), moderate (32.9–61.39) and high (> 61.39) resilience. Quantitative analysis showed that volunteers’ total resilience scores were predominantly in the moderate range (Table 1).

**TABLE 1:** Descriptive statistics of resilience scores among Indonesian Red Cross Demak volunteer (*N* = 69).

Measure	Mean	s.d.	Score range/cut-off	*n*	%
Total resilience score	47.14	14.24	20–77	-	100
Low resilience	-	-	< 32.9	15	21.7
Moderate resilience	-	-	32.90–61.39	44	63.8
High resilience	-	-	> 61.39	10	14.5

s.d., standard deviation.

### Qualitative results: Factors contributing to the disaster volunteer resilience

Volunteers having a high resilience level (Table 1) were purposively invited to the qualitative phase as main participants, complemented by external perspectives from the organisation (staff and board members) and volunteer’s families. Table 2 shows that qualitative participants were categorised based on role, age, gender and duration in disaster volunteering.

**TABLE 2:** Demographic data of the qualitative participants.

Participant	Role	Age (years)	Gender	Duration joins as volunteer/disaster activity (years)
P1	Volunteer	40	Male	19
P2	Volunteer	42	Female	9
P3	Volunteer	22	Female	6
P4	Volunteer	24	Female	11
P5	Volunteer	41	Male	8
P6	Volunteer	24	Female	7
P7	Volunteer	19	Male	3
P8	Volunteer	24	Female	3
P9	Organisation staff/board	52	Male	21
P10	Organisation staff/board	43	Male	5
P11	Organisation staff/board	62	Male	15
P12	Organisation staff/board	25	Female	8
P13	Organisation staff/board	34	Female	9
P14	Volunteer’s family	38	Female	-
P15	Volunteer’s family	45	Male	-
P16	Volunteer’s family	23	Female	-
P17	Volunteer’s family	24	Female	-

Note: Qualitative data were collected through face-to-face semi-structured individual interviews, and themes were derived using deductive and inductive approaches. Deductive analysis was guided by CD-RISC, while inductive analysis allowed themes from the data. Table 3 highlights the myriads of factors contributing to disaster volunteer resilience that inductively found in participants’ perceptions that were compared with the quantitative findings in the joint display framework.

**TABLE 3:** Joint display of Indonesian Red Cross Demak Volunteer resilience.

RQ: Theme group	Quantitative data	Qualitative data	Findings
Theme 1: Competency	Personal competence was among the highest-scoring CD-RISC dimensions, indicating strong perceptions of capability in managing challenges.	Competency emerged as the main theme, consisting of the sub-themes of competence in disaster response, active communication, and stress management. Personal competence illustrates how volunteers’ resilience in coastal disaster. This is reflected in the confidence in facing disaster challenges. This theme is supported by quantitative findings where scores related to the competency dimension are relatively high compared to others. Participants mention the importance of mastering technical disaster-response skills and updating knowledge regularly: *‘Surviving is not enough; we must also gain knowledge’* (P6, M, 41). Among women participants, resilience was supported and the conflict were managed through active communication with the family. Participant 2 explained regularly updating her location and activities during disaster assignment. Participant 1 described stress management competence as an important capacity enabled by training, time allocation to rest during assignment, humour, helping them ‘*avoid falling apart’* during operations. Participant 16 said that families encouraged open emotional expression to relieve pressure.	**Convergence**: Qualitative narratives reinforce the quantitative pattern that personal competence is a key resilience resource. Volunteers’ descriptions of skills, communication, and emotional regulation align with the high quantitative scores in this dimension.
Theme 2: Spirituality	Spirituality showed moderate mean scores relative to other dimensions.	Spirituality themes consisted of religious values, sincerity [*lilo legowo*], and humanitarian motivation sub-themes that encouraged them to persevere and view their duties as acts of worship. While quantitative scores placed religious values, sincerity, and humanitarian motivation lower than other dimensions, qualitative data revealed otherwise, stating that it helped volunteers endure challenges in the field. A participant agreed that they are doing the volunteer activity as *ibadah* [service] and a source of inner satisfaction: *‘Materially, there is nothing, but inner satisfaction comes from helping’* (P2, F, 42). Participants 5 and 15 mention the activity as a spiritual investment. And the Javanese value of *lilo legowo* [sincerity] was seen as follows: *‘To be resilient, we must be sincere above all else.’* (P2, F, 42). Participant 14 stated that families also emphasise that helping should be unconditional. Participant 4 said that humanitarian motivation also played a role, as volunteers balanced social service with self-reliance to ‘*keep helping others’, and* Participants 14 and 15 mentioned support by family members who valued time and energy given for collective good.	**Discordance:** The qualitative findings highlight spirituality as a deeply influential resilience resource, whereas the quantitative scores suggest only a moderate role. This discrepancy indicates that volunteers’ spiritual motivations and meaning-making processes may not be fully captured by the standardised CD-RISC spirituality items, revealing the importance of contextual, culturally grounded expressions of spirituality that exceed what numerical scores can quantify.
Theme 3: Culture	Cultural values are not included as indicators in the CD-RISC scale.	Culture emerges as the third theme consisting of the values of caring others [*ngopeni lan ngrumati*] and togetherness sub-themes. A participant explains the Javanese idiom ‘*Ngopeni lan ngrumati’* [caring and being considerate] especially during high-risk deployments as mutual support among PMI Demak volunteers. Furthermore, she emphasised the importance of maintaining each other’s commitment as volunteers because they understand: ‘*Volunteers are the frontliner*.’ (P13, F, 34). An understanding of ‘*collective resilience*’ emerges with shared activities carried out during assignments such as sharing meals, taking short breaks while on duty and working together to build solidarity (P1, M, 40). On the other hand, a participant said that: ‘*Serving in a disaster is a meaningful experience, especially being able to be together with other volunteers in difficult situations’* (P12, F, 25).	**Expansion:** Cultural values such as considerateness, togetherness, and solidarity although not captured in the quantitative resilience indicators emerged qualitatively as important contextual resources that strengthened collective support and volunteer morale.
Theme 4: Emotional support	Aspects of emotional regulation and interpersonal trust appear in the CD-RISC scores, but the scale does not directly assess family-based emotional support.	Obtaining emotional support factors as themes related to the ability to manage negative emotions, namely family empathic listening, family permission, and family understanding. Qualitative findings showed that emotional regulation was strongly supported by family relationships, particularly through empathic listening, permission, and understanding. Volunteers described feeling affirmed when families consistently encouraged participation: ‘They never discourage us’ (P1, M, 40)	**Expansion:** Qualitative findings extend the quantitative results by revealing that emotional support is not limited to individual abilities (e.g., emotion regulation) but also includes family-based relational support that is not captured by the CD-RISC items.
		One participant explained that permission from the family was given as long as the activity was something positive for the other person: ‘As long as the work is positive and you’re healthy, I’ll allow it.’ (P14, F, 38). Family understanding of disaster activities is an important key to family support and permission given to female volunteers. Support from the family is not only limited to permission but also helps provided, listening to the stories of volunteers, providing transportation support and offering other technical assistance during assignments.	
Theme 5: Functional support	In quantitative data, the dimension of functional support is not included in the resilience indicator.	Team-based collaboration, facilitation of volunteer needs during assignments, guidance from mentors, and post-assignment evaluation are part of functional support. Participant described team-based collaboration ensured continuity when members were absent: ‘*Everyone must be able doing their task in disaster*.’ (P2, F, 42) ‘‘*With backup from nearby units reinforcing collective strength*.’ (P11, F, 62). The organisation who deploys volunteers provides basic needs during deployments, provided equipment, lodging, and stress-management training to support readiness: ‘*They already work voluntarily, and when they’re hungry, it’s a pity*.’ (P10, M, 43) Participants 1, 7 and 16 all stated that mentorship was another valuable form of support, with experienced members offering guidance, reminders, and motivation in both school-based and field settings. Finally, Participant 10 stated that post-assignment evaluations allowed teams to reflect and adjust for future responses, often combined with informal meals to rebuild team morale.	**Expansion:** Qualitative data reveal organisational and team-based support as vital resilience resources not represented in quantitative measures.

Note: *RQ 2*: Factors contributing to Disaster Volunteer Resilience.

PMI, Indonesian Red Cross; CD-RISC, Connor–Davidson Resilience Scale; M, male; F, female.

## Discussion

Exploring disaster volunteer resilience in coastal areas is essential for enhancing their motivation, knowledge and skills to counteract the negative impact of mental problems during involvement in disasters as well as improving disaster mitigation. This mixed methods study explores resiliency using a multisystem that combines internal and external perspectives at the coastal context of low-county and middle-county prone to disaster. This study has two main goals: To quantitatively assess volunteer resilience using the adapted Connor-Davidson Resilience Scale and to qualitatively explore the factors contributing to disaster volunteer resilience, specifically in a rural–peri-urban coastal district of Central Java, Indonesia, following the 2024 Demak floods. Moderate levels of resilience were reported across the five dimensions, specifically in the personal competence and internal trust dimensions record higher mean scores than the others. The qualitative findings further explicate how this multisystem resilience is enacted in practice, consistent with previous Indonesian disaster volunteer resilience study (Dewi et al. 2025a). The five themes, ‘volunteer competencies, spirituality, culture, emotional support, and functional support’, indicate that disaster volunteer resilience is better understood as a multisystem capacity embedded in everyday collective or communal relationships with families, organisations and communities rather than as an individual trait alone (Connor & Davidson 2003; Sanson & Masten 2024; Ungar et al. 2021a). These findings align with prior research showing that volunteer resilience emerges from the interaction of individual, environmental and organisational (Ghodsi et al. 2020). However, this study provides evidence that is context specific on the factors that are expressed among affiliated volunteers responding to a recurrent coastal flood disaster in Central Java. In a recent review, Ghodsi et al. (2020) found limited studies on volunteer resilience, most of which focused on humanitarian workers as well as expatriate professionals in high-income contexts. The latest review has similarly noted that disaster volunteer resilience remains underexplored, particularly in non-Western and low- and middle-income settings. By contrast, this study contributes to an emerging body of research in Southeast Asia, showing how spiritual commitment, family consent and collective values help sustain volunteer motivation (Arianti et al. 2025; Ruslanjari et al. 2024). Though contexts differ which Demak’s tidal floods are slow-onset and recurring, unlike sudden earthquakes, volunteers in both settings drew on relational and moral resources to interpret their roles and regulate emotional strain. Personal competence emerged as a particularly salient resilience resource in both quantitative and qualitative studies. In the quantitative findings, this dimension showed relatively stronger prominence; in the qualitative accounts, competence was expressed through technical preparedness, active communication and stress management during deployment. This suggests that competence in disaster volunteering is not only a matter of confidence or task mastery but also of maintaining communication, managing emotional strain and learning from field experience. This interpretation is consistent with the literature, suggesting that adaptive capacity develops through experiential learning, mentoring and reflective practice rather than being fixed as an individual trait (Liu, Reed & Fung 2020; Masten 2021; Southwick et al. 2014). This finding is also compatible with Masten’s view of resilience as ‘ordinary magic’, in which adaptation is supported by ordinary protective systems and with later multisystem perspectives that locate resilience across interacting individual, relational and community systems (Masten 2001, 2014). In this study, individual resilience capacities such as personal competence and internal trust were enhanced by external support. This relational perspective is also reflected in recent ecological and multisystem models of resilience (Ungar 2021; Ungar et al. 2021a). In this sense, resilience is not best understood as a fixed property of the volunteer, but as a context-sensitive capacity supported by the alignment of internal strengths and surrounding systems (Liu et al. 2020). Emotional support, especially from family members, was another important contributing factor. In this study, emotional support took the form of empathic listening, permission, understanding and practical assistance from relatives. These findings suggest that resilience among affiliated volunteers is not only a matter of individual emotional regulation but also of relational regulation, in which family members help volunteers manage stress, sustain participation and negotiate role demands. A relational well-being lens discusses the emergence of family and organisational supports (White & Jha 2023). Material provisions, including meals and equipment, interacted with relational care (e.g. relatives’ attentiveness, peer support) and cultural narratives to foster a sense of purpose. For women, negotiated domestic arrangements facilitated participation in high-risk assignments without affecting family duties. These relational factors retained dignity, purpose and motivation at prolonged deployments, suggesting that resilience was collectively generated through close family and social interactions (Boon et al. 2012; Southwick et al. 2014).

Two themes that extended the quantitative profile were cultural value and spirituality. Cultural values such as ‘*ngopeni lan ngrumati*’ [mutual care], togetherness and solidarity emerged as important resilience resources even though they were not directly represented in the quantitative indicators. This corresponds to research from Southeast Asia that conceptualises disaster coping as relational and spiritually integrated (Hechanova & Waelde 2017). Our research underscores that, for Javanese volunteers, resilience encompasses the fulfilment of collective responsibilities grounded in cultural and religious contexts. Spirituality was a major source of meaning-making (Pargament & Mahoney 2016). Volunteers regarded their activities as *ibadah* [worship], and the Javanese term *lilo legowo* [sincere acceptance] facilitated their perception of pain as a moral obligation. In this instance, spirituality functioned as more than a private coping mechanisms; it was relational, with families, organisations and cultural narratives together fostering a collective comprehension of sacrifice and service (Hakkim & Deb 2022; Sato et al. 2022). The mixed methods design was particularly important because it showed that several resilience resources central to volunteers’ adaptation were only partially captured, or not captured at all, by individual-level quantitative indicators. In this study, the adapted CD-RISC was useful for describing resilience levels and identifying participants with relatively high resilience scores. However, the qualitative findings showed that some important contributing factors, especially cultural values, family-based emotional support and organisational functional support, became more visible only when resilience was explored in context. This is consistent with arguments that resilience measurement is closely tied to how resilience is conceptualised, and that individual-level scales may not fully capture broader socio-ecological sources of adaptation (Liu et al. 2020; Windle 2011). Accordingly, the present study demonstrates the value of interpreting resilience scores alongside qualitative accounts of the broader social ecology in which resilience is sustained. The practical implications are reflected through functional support that emerged as a theme in this study, including team-based collaboration, facilitation of volunteers’ needs during deployment, guidance from mentors and post-assignment evaluation. These supports were described as helping volunteers remain effective, feel cared for and continue their engagement during prolonged disaster response. Previous studies likewise show that organisational factors such as role clarity, preparation, training, assigned tasks and support are closely related to volunteer mental health and resilience (Asrofi, Giyarsih & Hadmoko 2024; Thormar et al. 2013). During the 2024 response, over 100 PMI Demak personnel were deployed, with volunteers providing food, medical and psychosocial services to affected communities. Yet spontaneous arrivals and limited coordination created stress for both volunteers and staff. Previous studies have noted similar barriers in disaster-prone regions, including organisational capacity constraints and insufficient preparedness (Asrofi et al. 2024; Dinas Komunikasi dan Informatika Kabupaten Demak 2024). Strengthening institutional systems for volunteer management, including preparation, psychosocial debriefing and family-informed protocols that remain a priority. A key strength of this study lies in its sequential explanatory mixed methods design, which enabled an integrated examination of disaster volunteer resilience through both quantitative measurement and qualitative exploration. The near-census quantitative sample enhanced the descriptive robustness of the resilience profile, while the inclusion of volunteers, family members and organisational staff in the qualitative phase allowed for multisystem triangulation of resilience processes. The use of a joint-display technique further strengthened analytic integration by explicitly linking quantitative indicators with qualitative themes.

### Study limitation

This study focused on volunteers from a single organisation within one disaster context, which may limit the transferability of the findings. In addition, the qualitative phase involved participants with relatively higher resilience scores, which was appropriate for identifying contributing factors but may not capture the experiences of volunteers with lower resilience. Future research would benefit from longitudinal or intervention-based designs that examine how personal, relational, organisational, cultural and spiritual resources interact over time across diverse disaster-prone settings.

## Conclusion

This study found that volunteers involved in the 2024 Demak flood response generally demonstrated moderate levels of resilience, with personal competence and internal trust emerging as relatively stronger domains in the CD-RISC profile. The thematic analysis further showed that disaster volunteer resilience in this recurrent coastal flood context was sustained through five interrelated themes: Personal competence, spirituality, culture, emotional support and functional support. Taken together, these findings suggest that resilience was shaped not only by individual coping alone but also by the interaction of individual, relational, organisational and cultural resources. In particular, the qualitative findings indicated that volunteers’ capacities were reinforced through family permission and empathic listening, team-based collaboration, organisational support and culturally grounded practices of spirituality, togetherness and *ngopeni lan ngrumati*. These findings suggest that efforts to strengthen affiliated disaster volunteer resilience should extend beyond individual coping skills to include family-inclusive, culturally sensitive and organisationally supportive environments.

The research findings have the following practical and specific implications:

- For policymakers in disaster management: National Disaster Management Agency or BNPB, Regional Disaster Management Agency or BPBD, it is necessary to clearly identify disaster volunteer resilience as an essential element of disaster risk reduction and emergency response. Policies and operational guidelines must foster multisystem resilience by integrating individual capacity development with family, organisational and culturally grounded resources, especially during extended and recurring climate-related disasters. To keep local volunteer capacity over time, it is important to strengthen bottom-up, context-sensitive approaches.
- For humanitarian and civil society organisations: Organisations that utilise disaster volunteers (such as the PMI) may come up with strategies for building resilience that extend beyond technical competence to include actions, including mentoring, organisational procedures and team-based coordination that strengthen both individual and group resilience. Recognising cultural values, shared spirituality and functional support as resilience resources may strengthen volunteers’ adaptive capacity and maintain engagement during prolonged disaster response.
- For future researchers: Disaster volunteer resilience enhancement programme should be further researched through longitudinal and intervention-based methodologies to analyse the evolution of multisystem resilience processes over time. It needs to modify resilience measurement tools in order to better reflect relational, cultural and spiritual dimensions, particularly in low- and middle-income and climate-vulnerable settings.
